# Enhanced Magnesium Ion Sensing Using Polyurethane Membranes Modified with ĸ-Carrageenan and D2EHPA: A Potentiometric Approach

**DOI:** 10.3390/bios16010055

**Published:** 2026-01-12

**Authors:** Faridah Hanum, Salfauqi Nurman, Nurhayati, Nasrullah Idris, Rinaldi Idroes, Eka Safitri

**Affiliations:** 1 Graduate School of Mathematics and Applied Sciences, Universitas Syiah Kuala, Banda Aceh 23111, Indonesia; faridah63@mhs.usk.ac.id; 2 Department of Pharmacy, Poltekkes Kemenkes Aceh, Aceh Besar 23231, Indonesia; 3 Department of Marine Engineering, Politeknik Pelayaran Malahayati, Aceh Besar 23381, Indonesia; salfauqi@poltekpelaceh.ac.id; 4 Department of Pharmacy, Faculty of Health Sciences, Universitas Ubudiyah Indonesia, Banda Aceh 23114, Indonesia; nurhayati@uui.ac.id; 5 Department of Physics, Faculty of Mathematics and Natural Sciences, Universitas Syiah Kuala, Banda Aceh 23111, Indonesia; nasrullah.idris@usk.ac.id; 6 Department of Pharmacy, Faculty of Mathematics and Natural Sciences, Universitas Syiah Kuala, Banda Aceh 23111, Indonesia; rinaldi.idroes@usk.ac.id; 7 Department of Chemistry, Faculty of Mathematics and Natural Sciences, Universitas Syiah Kuala, Banda Aceh 23111, Indonesia

**Keywords:** Mg^2+^ ISE, potentiometric, polyurethane membrane, ĸ-carrageenan, D2EHPA, hydrophilicity, cross-linking, ∆E stability

## Abstract

Magnesium (Mg^2+^) ions require sensitive and selective detection due to their low concentrations and coexistence with similar ions in matrices. This study developed a potentiometric ISE using a new modified polyurethane membrane. The membrane’s negative surface charge facilitates selective interaction with Mg^2+^ ion. Optimal performance was obtained at 0.0061% (*w*/*w*) κ-carrageenan and 0.0006% (*w*/*w*) D2EHPA. The ISE exhibited a near-Nernstian response of 29.49 ± 0.01 mV/decade across a 10^−9^–10^−4^ M concentration range (*R^2^* = 0.992), with a detection limit of 1.25 × 10^−10^ M and a response time of 200 s. It remained stable in the pH range 6–8 for one month and demonstrated high selectivity over K^+^, Na^+^, and Ca^2+^ ($K_{ij}$ < 1). The repeatability and reproducibility tests yielded standard deviations of 0.15 and 0.39, while recovery rates confirmed analytical reliability. The water contact angle analysis showed a reduction from ~80° to ~69° after membrane conditioning, indicating increased hydrophilicity and improved interfacial for ion diffusion. FTIR analysis confirmed successful modification by reduced O–H peak intensity, while XRD verified the amorphous structure. SEM revealed a dense top layer with concave morphology, favorable for minimizing leakage and ensuring efficient ion transport within the sensing system.

## 1. Introduction

Various studies and research on magnesium (Mg), one of the essential main elements involved in normal biological functions, such as potassium (K), sodium (Na), and calcium (Ca), have emerged as an interesting topic in recent times [1]. The Mg^2+^ ion is not needed in large amounts, but the deficiency might lead to cystic fibrosis [2], neurodegenerative manifestations such as Alzheimer’s and Parkinson’s [1,3], malnutrition, various syndromes, diabetes mellitus [4], and infectious diseases such as coronavirus disease-19 (COVID-19) [5]. Number issues are suspected to cause imbalances in Mg levels in the body, which is not only related to personal health but also has a broader impact on global public health. This finding underscores the importance of monitoring Mg levels in medical samples to accurately interpret the pathological state of physiological processes. This is also a reason to examine Mg intake from food and medicine. Consequently, the analysis of Mg becomes an interrelated regulation.

Several techniques, such as atomic absorption spectroscopy [6], nuclear magnetic resonance [6,7], and X-ray fluorescence [8], have been used for Mg^2+^ detection. These techniques can detect samples simultaneously but involve complicated instrumentation and require trained operators. Therefore, simpler alternative methods with sensitivity and accuracy are still needed. In addition, Mg^2+^ ions are often present at low levels of free-ion concentrations or coexist with other ions in various samples, requiring precise analytical methods for sensitive and accurate Mg detection [9].

Potentiometry uses an ion-selective electrode (ISE) and consists of a selective membrane determined by an active substance. The development of modified ISE membranes for Mg^2+^ ion detection has advanced significantly through the introduction of new materials. A number of Mg^2+^ ISEs have been developed using a thin-film photopolymer of Poly(Tetrahydrofurfuryl Acrylate) (pTHFA). The ISE is able to detect Mg^2+^ ion concentrations of 10^−6^ to 10^−1^ M with a Nernstian response of 26.9 mV/decade [10]. Ahmed S Saad et al. [11] developed a sensor using esomeprazole magnesium trihydrate with a sensitivity approaching Nernstian 29.93 mV/decade in the concentration range of 1.41 × 10^−5^–1 × 10^−2^ M, and a detection limit of 4.13 × 10^−6^ M. Previously, an Mg^2+^ sensor has also been developed using Extended Gate Field Effect Transistor (EGFET) based on a silicon membrane with a sensitivity of 31.75 mV/decade in the concentration range of 10^−4^–1 M [12]. Although these developed Mg^2+^ sensors have sensitivities close to Nernstian and their response is linear to Mg^2+^ ion levels in most samples, the actual response is still limited by the presence of Ca^2+^ ions. It might be due to the competition between Mg^2+^ and Ca^2+^ ions for the ionophore or ionic carrier [13,14,15,16]. Controlling these drawbacks challenges experts to explore new suitable materials and specific ionophores. The membrane and active substances work synergistically to produce an actual signal corresponding to the analyte, enhancing sensitivity and selectivity.

The polyurethane (PU) membrane is the main focus of our research, and its progress remains slow. This membrane is known for excellent mechanical properties, chemical stability, and ease of modification for specific functions, including ion sensors. Previous studies have reported the successful modification of PU membranes by incorporating cerium (IV) phosphate, synthesized using Ce(SO_4_)_2_·4H_2_O as a precursor, to enhance their ion-exchange capacity—an essential characteristic for ion separation and sensing applications. In particular, Baig and Khan demonstrated that a composite membrane with a 1:2 ratio of PU/cerium (IV) phosphate exhibited the best ion-exchange capacity and performed effectively as a simple, low-cost potentiometric sensor for Cu^2+^ ion detection [17]. Therefore, the study of developing PU membranes functioning as sensor matrices remains an important research challenge.

The PU membrane is a type of charged membrane because it contains urethane groups with negatively charged carbonyl groups. This condition allows the PU membrane to bind with positive ions and can be used as a metal-sensitive membrane [18,19]. Further modification is necessary to enhance its properties as a sensor matrix. The membrane was synthesized from castor oil (*Ricinus communis* L.) and toluene-2,4-diisocyanate (TDI), and the modified membrane was employed as a matrix to detect Pb^2+^ ions using 1,10-phenanthroline [19], but this sensor lacks stability.

In this study, the PU membrane was further modified with κ-carrageenan containing sulfate functional groups to facilitate the formation of strong cross-links and enhance the membrane’s negative charge density. Biological components are widely used in the development of sensing membranes and constitute key elements of bio-inspired sensing interfaces. In addition, di-(2-ethylhexyl) phosphoric acid (D2EHPA) has been widely used as a carrier in liquid membrane systems, exhibiting a high affinity toward divalent metal ions to form reversible coordination complexes through its phosphate groups. The functionality facilitates the selective transport of ions across the membrane, providing active binding sites for the target analyte [20]. The interfacial equilibrium established at the membrane surface generates a potential that is directly proportional to the concentration of analyte. This study presents the first instance of a dual-functionalized PU membrane integrating both κ-carrageenan and D2EHPA for magnesium ion potentiometric detection. This novel approach demonstrates enhanced selectivity, sensitivity, and stability in the detection.

## 2. Materials and Methods

### 2.1. Materials

All chemicals used in this work were prepared with analytical grades from Merck, including acetone, 2,4-toluene diisocyanate (TDI), D2EHPA, magnesium nitrate (Mg(NO_3_)_2_), sodium nitrate (NaNO_3_), potassium chloride (KCl), potassium nitrate (KNO_3_), calcium nitrate (Ca(NO_3_)_2_), iron (III)-chloride (FeCl_3_), phosphate buffer, lithium acetate (CH_3_COOLi), and silver (Ag) wire.

Commercial castor oil (*Ricinus communis* L.) and ĸ-carrageenan were procured from PT. Rudang Jaya (Medan, Indonesia) and were of industrial-grade quality. Agar was purchased from the trademark Akos. A centrifuge tube was employed as the electrode based on its favorable properties and compatibility with the measurement setup. A nitrile butadiene rubber (NBR) O-ring was employed exclusively as a mechanical sealing component to fix the thin PU membrane at the mouth of the electrode, without contributing to the electrochemical response of the electrode system.

### 2.2. Instruments

Instruments used here for membrane characterization included an FTIR spectrophotometer (Shimadzu Prestige, Kyoto, Japan), XRD (Shimadzu XRD-700 Series, Kyoto, Japan), HT8503 Universal Testing Machine (Hung Ta Instrument Co., Ltd., Taichung, Taiwan), and scanning electron microscope (SEM; Jeol Jsm 6360 LA, Tokyo, Japan). The water contact angle (WCA) was measured using a Krüss DSA25 goniometer (Krüss GmbH, Hamburg, Germany). The analytical measurements were performed using an Orion potentiometer (Thermo Orion Scientific Star A2115; Waltham, MA, USA), equipped with a digital benchtop pH meter. A handmade ISE and Ag/AgCl reference electrode were also used to investigate the sensing ability of the electrodes, which were synthesized according to Ref. [18].

### 2.3. Preparation and Construction of PU-Based Sensing Membrane

**Scheme 1:**
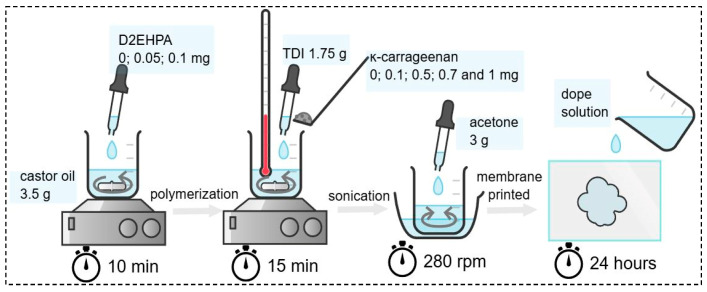
Synthesis of modified polyurethane membrane (created in Chemix.org).

The dope solution formulations were prepared at the following combination ratios: D2EHPA, castor oil, κ-carrageenan, TDI, and acetone. The membrane composition refers to Scheme 1, which is based on the average optimized ratio as reported in a previous study [21]. Initially, D2EHPA was blended with castor oil and stirred until the mixed solution was homogeneous. Subsequently, κ-carrageenan was incorporated under continuous stirring at 40 °C, followed by the polymerization process initiated by the addition of 1.75 g of TDI at the same temperature. As much as the addition of 3 g of acetone was to eliminate entrapped air bubbles before casting onto a glass substrate. The membrane was then allowed to dry at ambient conditions for 24 h. The membrane thickness was kept constant using a fixed casting volume and identical drying conditions.

For ISE assembly, the prepared PU-based sensing membrane was mounted at the end of a plastic centrifuge tube and sealed with an O-ring. The electrode was filled with an internal Mg^2+^ solution at the selected concentration. A handmade Ag/AgCl reference electrode, prepared using a silver wire and KCl solution, was inserted into the internal solution and electrically connected to the potentiometer, following the procedure reported in Ref. [18].

### 2.4. Mg^2+^ ISE Optimization

The Mg^2+^ ISE was optimized by evaluating sensitivity and linearity, which initially vary in the κ-carrageenan to D2EHPA ratio. Subsequently, internal solution compositions were prepared by varying Mg^2+^ concentrations in 0.1 M KCl, and the electrode potential was measured against standard Mg^2+^ solutions (10^−10^–10^−1^ M). Calibration curves were plotted for electrode potential (mV) versus Mg^2+^ ion concentration of –log [Mg^2+^] (M). A 10^−3^ M NaNO_3_ TISAB solution was employed to maintain constant ionic strength, such that the activity coefficient of Mg^2+^ could be kept constant over the concentration range. Therefore, concentration-based calibration reliably represents the electrode response and enables accurate determination of sensitivity, linear range, LoD, and LoQ [18].

Prior to all measurements, the ISE membrane was conditioned in a 0.1 M standard Mg^2+^ solution for 24 h, followed by rinsing with distilled water. Potentiometric readings were recorded only after the potential reached a stable condition (≤±1 mV), which was typically achieved after approximately 120 s, ensuring reproducible and reliable potential measurements.

### 2.5. Mg^2+^ ISE Performance Evaluation

The performance evaluation of the ISE was conducted by examining several critical parameters to ensure its analytical reliability. The LoD was determined based on the potential response of a blank solution containing 10^−3^ M NaNO_3_, calculated as the mean blank signal plus three times its standard deviation (SD) [22]. In parallel, the LoQ was established as the lowest concentration within the well-defined linear range that provided a reliable and reproducible potential response, corresponding to the blank signal plus ten times the SD [23].

The effect of pH on ISE stability response was determined using a standard solution of 10^−4^ M (Mg(NO_3_)_2_) in 10^−1^ M phosphate-buffered solutions (pH 4–9) [18]. Selectivity ($K_{ij}$) was assessed by the Separate Solution Method (SSM) using equimolar solutions of Mg^2+^ and the interfering ions K^+^, Na^+^, and Ca^2+^.

The response time was evaluated using standard working solutions within the ISE’s linear range. The potentials (mV) were continuously recorded at 30 s intervals to assess the time required to reach a steady response. Repeatability and reproducibility were evaluated using 10 independently fabricated Mg^2+^ ISEs within the linear concentration range. Repeatability was assessed via six consecutive measurements per electrode, expressed as mean potential ± SD, and the relative standard deviation (%RSD) was calculated as (SD/mean) × 100. Reproducibility was determined from the variation of the average potentials obtained across all electrodes, with %RSD likewise employed as a quantitative indicator of measurement precision [23]. Electrode lifetime was evaluated for 33 days by monitoring the ISE’s sensitivity and determining deviations from the theoretical Nernstian slope for divalent ions (29.58 mV/decade).

The recovery test measured the ISE’s potential response in several standard solutions, randomly selected within the linear concentration range. The corresponding concentrations (x-values) were obtained by interpolating the measured potentials using the regression equation of the previously established calibration curve. These calculated concentrations were then compared to the known concentrations to determine the recovery percentage.

## 3. Results and Discussion

### 3.1. Modified Polyurethane Membrane Characterizations

The functional group of the synthesized PU membrane was initially analyzed using FTIR spectroscopy to identify chemical interactions generated by the incorporation of κ-carrageenan and D2EHPA into the PU matrix. The crystallinity characteristics of the membrane were evaluated via XRD to quantify the degree of structural order enhanced by these modifications. Surface morphology was further investigated using SEM to examine physical properties related to ion transport. These comprehensive characterizations were performed to confirm the effectiveness of the membrane modification process and determine its potential performance as a selective matrix for Mg^2+^ ion detection.

**Figure 1:**
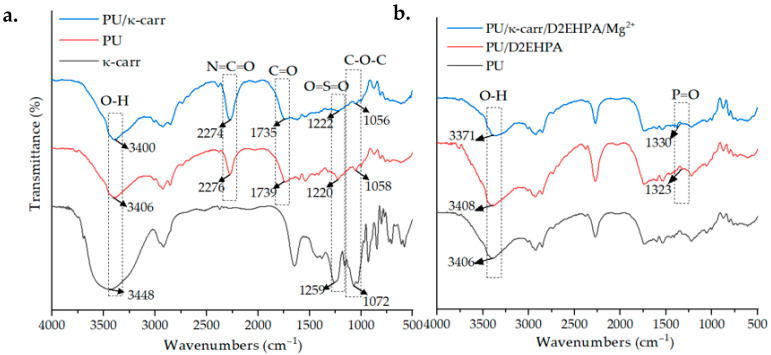
FTIR spectra of modified polyurethane membranes. Interaction of κ-carrageenan showing cross-link (**a**) and interaction of D2EHPA indicating dimerization and complexation (**b**) during matrix of Mg^2+^ ISE preparations.

The FTIR spectra of κ-carrageenan, PU, and PU/κ-carr membranes are shown in Figure 1a. A broad O–H stretching band observed at 3448 cm^−1^ in κ-carrageenan shifted to 3406 cm^−1^ in PU and 3400 cm^−1^ in PU/κ-carr, indicating modified hydrogen bonding due to polyurethane formation. Urethane linkages (–NH–COO–) are formed by the interaction of hydroxyl groups from castor oil and κ-carrageenan with isocyanate groups from TDI, as confirmed by strong C=O stretching vibrations at 1739 cm^−1^ and 1735 cm^−1^, respectively. These pathways provide the principal framework of the cross-linking network inside the PU structure [24,25]. The polymerization at 40 °C is expected to enhance the reactivity of the isocyanate group, accelerating cross-linking via urethane addition without generating byproducts [26]. The N=C=O absorption bands at 2276 cm^−1^ (PU) and 2274 cm^−1^ (PU/κ-carr) indicate residual isocyanate groups, as supported by previous findings [18,19,27]. This may be due to an imbalanced –OH/–NCO stoichiometric ratio, in which the excess has been associated with enhanced hydrophobicity of the PU [26]. The presence of bands O=S=O (1270–1230 cm^−1^) and C–O–C (1100–1000 cm^−1^) indicates that the basic structure of the polymer is preserved, while also confirming the presence of hydrophilic functional groups such as sulfate and ether, which are responsible for the inherent water affinity of κ-carrageenan [19,26].

Figure 1b illustrates comparisons of the FTIR spectra for PU, PU/D2EHPA, and PU/κ-carr/D2EHPA/Mg^2+^ membranes. Absorption bands for O–H and P=O were identified at around 3570–3200 cm^−1^ and 1250–1350 cm^−1^, respectively. The collective shift of the broadened O–H band and the P=O stretching vibration to higher wavenumbers in the PU/κ-carr/D2EHPA/Mg^2+^ spectrum indicates strong hydrogen bonding and coordination with Mg^2+^ ion, suggesting the formation of metal–ligand complexes. These findings confirm that D2EHPA in non-polar solvents (i.e., castor oil) undergoes a transition from monomers to dimers as a complex form [28]. Its function as a selective carrier has been proven in a wide range of metal ion extraction applications and detection systems, including ESI [16,19,26,29]. The lipophilic characteristic and the availability of lone electron pairs on the anionic phosphate groups enable the D2EHPA to form reversible interactions with divalent metal ions, producing a significant potential gradient in membrane sensor applications.

**Figure 2:**
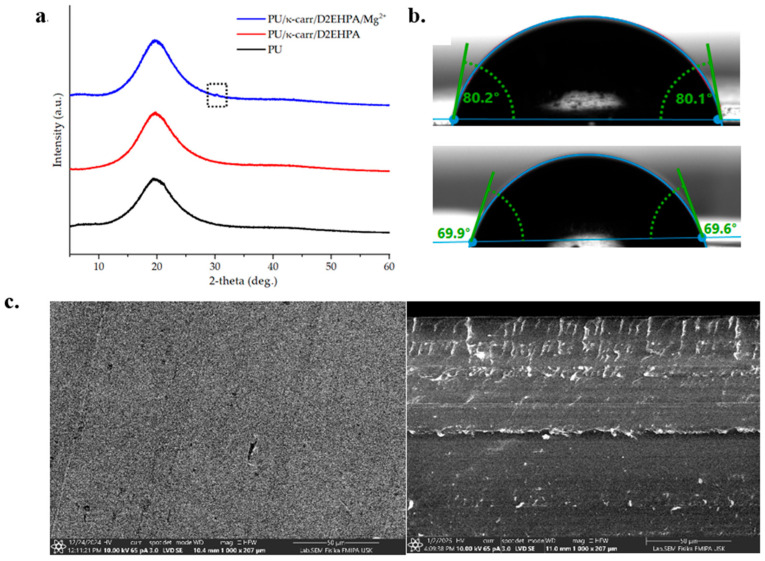
Physical characterization results of membranes. XRD patterns of PU, PU/κ-carrageenan/D2EHPA and PU/κ-carrageenan/D2EHPA/Mg^2+^ membranes (**a**); WCA measured on the surface of PU/κ-carrageenan/D2EHPA membranes before and after conditioning (**b**); SEM image of PU/κ-carrageenan/D2EHPA/Mg^2+^ membrane surface and cross-section (**c**).

The modified PU membranes exhibited crystalline properties, as shown in Figure 2a. All membrane samples exhibited broad diffraction peaks, indicating their predominantly amorphous nature. This observation was consistent with previous findings [18,26]; the incorporation of κ-carrageenan did not induce crystallinity, even after urethane cross-linking with isocyanate groups. The lack of sharp or distinct diffraction features further suggested that both κ-carrageenan and D2EHPA were homogeneously distributed within the membrane matrix [29]. Notably, the PU/κ-carr/D2EHPA/Mg^2+^ membrane showed a slight increase in peak intensity compared to the undoped PU/κ-carr/D2EHPA system. This enhancement was attributed to the interaction between Mg^2+^ ions and the polar functional groups of the membrane, which likely promoted localized ordering within the polymer network. A minor but distinct diffraction signal appeared at 2θ = 30.3°, as marked in the dotted box in the figure, potentially indicating the formation of Mg^2+^-induced microdomains or weak semi-crystalline structures. A similar trend was previously reported in other PU–metal ion coordination systems [17].

The water contact angle (WCA) characterization (Figure 2b) revealed that the modified PU membrane without Mg^2+^ ion preconditioning exhibited a WCA of approximately 80° [30], corresponding to an almost hydrophobic behavior. Such interfacial properties restrict the wetting of the membrane, hinder ion diffusion toward the electrode surface, and consequently increase the susceptibility to fouling due to the limited formation of a protective hydration layer. In contrast, Mg^2+^ ion conditioning for 24 h reduced the WCA to ~69°, indicating improved hydrophilicity that facilitates ion transport and provides a surface environment less prone to fouling. These findings are consistent with Mani et al. [31], who demonstrated that Mg-enriched PU matrices significantly enhance wettability, strengthen interfacial interactions, and mitigate biofouling. Similarly, Safitri et al. [18] emphasized that the engineering of membrane interfacial properties plays a critical role in lowering diffusion resistance and ensuring stable performance in ionic media. These results suggest that Mg preconditioning is an effective strategy to tailor the interfacial characteristics of PU membranes, thereby reducing fouling propensity and improving their applicability as ISE matrices.

In line with the XRD results, SEM analysis (Figure 2c) revealed that the modified membrane exhibited enhanced rigidity compared to pure carrageenan, confirming successful chemical modification [26]. Post-coordination with Mg^2+^ ions, the membrane maintained a dense and homogeneous morphology. These results are related to previous reports [17,18]. The surface displayed a compact outer layer with internal concavity, a structure favorable for preventing leakage while promoting efficient ion transport—critical for sensor functionality.

### 3.2. Mg^2+^ ISE Electrochemical Performance

A total of 13 membrane compositions were evaluated in terms of sensitivity, measurement range, and linearity using a fixed internal solution of Mg(NO_3_)_2_:KCl (0.1:0.1 M). This study led to the optimum matrix performance (Table 1).

An appropriate membrane composition was utilized to assess the optimal internal solution, while the influence of TISAB presence on ISE sensitivity has been reported to be significant [18].

**Figure 3:**
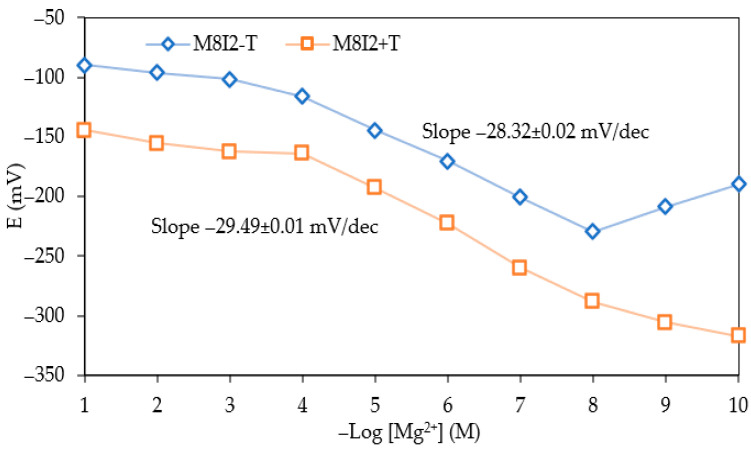
Potential response of Mg^2+^ ISE comparison between optimum membrane matrix sensor and internal solution, with and without TISAB.

This study confirmed that the Mg^2+^ ion sensor matrix containing 0.0061% ĸ-carrageenan and 0.0006% D2EHPA (*w*/*w*) obtained an ideal sensitivity of 29.49 ± 0.01 mV/decade in the concentration range of 10^−9^–10^−4^ M and a determination coefficient of 0.9919. Comparison of sensitivity values based on the potential response of Mg^2+^ ISE optimal conditions (denoted as M8I2+T) with no TISAB (denoted as M8I2–T) is presented in Figure 3.

Figure 3 exhibits a linear potential response of Mg^2+^ ISE under controlled ionic strength conditions. The incorporation of 10^−3^ M NaNO_3_ TISAB produces a near-Nernstian response of −29.49 ± 0.01 mV dec^−1^. In the absence of TISAB, the slope decreases to −28.32 ± 0.02 mV dec^−1^ and gives unstable responses at higher Mg^2+^ ion concentrations, indicated by relatively high SD. It might be attributed to uncontrolled variations in ionic strength and activity coefficients.

Comparison of membrane composition, internal solution variation, and TISAB to sensitivity values, measurement pathways, and Mg^2+^ ISE determination coefficients are shown in Table 1, Table 2 and Table 3. The best-performing Mg^2+^ ISE (denoted as Mg^2+^|M8I2+T ISE) in this study resulted in a LoD of 1.25 × 10^−10^ M and a LoQ of 1.09 × 10^−10^ M (Figure S1). In the ISE system, the detection limit is determined by the active ingredients in the membrane [32].

**Scheme 2:**
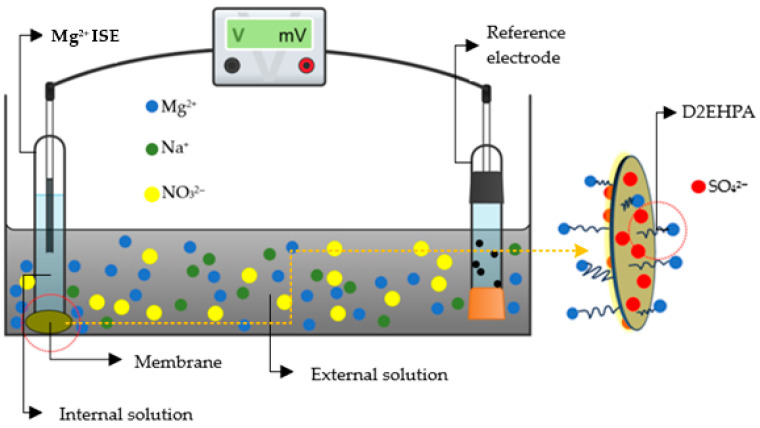
Facilitated counter-ion transport in the Mg^2+^ ISE system (created in Chemix.org). The yellow dashed line indicates the membrane interfacial coordination mechanism.

Accordingly, a higher ion-binding specification of the active side to the analyte enables improved detection at low concentration levels. In this study, the formation of a specific coordination complex between D2EHPA and Mg^2+^ is a key factor contributing to the enhanced sensor response. The modified PU/κ-carrageenan membrane promotes sulfate (SO_4_^2−^) groups that electrostatically attract Mg^2+^ ions and facilitate their subsequent coordination with D2EHPA. The synergistic role of these two modifiers in promoting facilitated counter-ion transport through the system is illustrated in Scheme 2, while the corresponding molecular interactions and their functional implications in the Mg^2+^ ISE system are summarized in Table 4.

The concentration gradient of Mg^2+^ ions between the internal filling solution and the external solution drives initial ion diffusion toward the membrane, in agreement with Le Chatelier’s principle, which predicts the migration of chemical species from regions of higher to lower concentration to re-establish equilibrium [33]. At the membrane interface, coordination-driven selectivity is established through electrostatic and coordinative interactions between D2EHPA molecules and Mg^2+^ ions, forming transient complexes via the oxygen atoms of the phosphate groups, while NO_3_^−^ anions are excluded due to electrostatic repulsion. This selective interfacial coordination promotes facilitated Mg^2+^ transport across the membrane. The strong affinity of D2EHPA toward Mg^2+^ ensures specific ion recognition and efficient charge transfer, enabling stable and sensitive potential responses even at very low ion concentrations, consistent with interfacial coordination mechanisms reported by Safitri et al. [18]. This phenomenon is reflected in a reduced LoD, indicating enhanced capability for trace Mg^2+^ sensing. Moreover, the formation of these coordination complexes amplifies the potential response to small variations in Mg^2+^ concentration, resulting in a significant increase in sensor sensitivity [34,35]. Subsequently, κ-carrageenan contributes to membrane charge stabilization and facilitates the transport process through electrostatic preconcentration and counter-ion support within the membrane matrix, as discussed in Scheme 2 and summarized in Table 4.

### 3.3. The pH Effect and Selectivity Toward Mg^2+^ Ion

**Figure 4:**
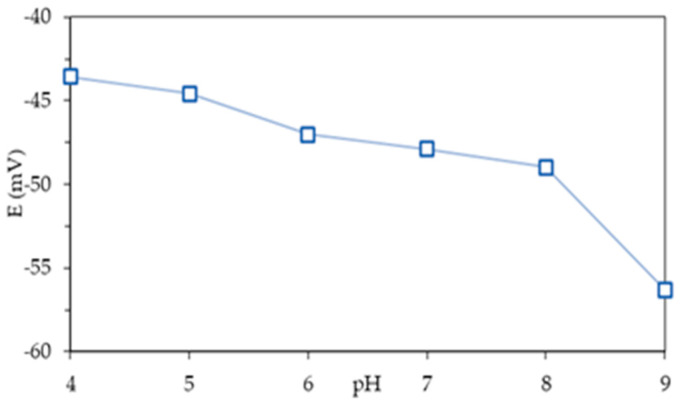
Potential response of the Mg^2+^|M8I2^+^T ISE as a function of pH effect in 10^−4^ M (Mg(NO_3_)_2_).

The Mg^2+^|M8I2+T ISE potential in this study exhibits an increasing response in the pH range of 4–5 while remaining steady in the pH range of 6–8 (Figure 4). Elevated H^+^ concentrations lead to membrane protonation, hence disrupting the potential [36]. This might be due to competition between Mg^2+^ and H^+^ ions that disrupt measurements, leading to a rise in potential at low pH. At pH 9, the potential response goes down because the metal hydroxide complex Mg(OH)_2_ is formed, which lowers the concentration of Mg^2+^ ions in solution.

The selectivity data for the Mg^2+^|M8I2+T ISE was acquired by assessing the potential of the primary ion, Mg^2+^, alongside analogous ions present in urine, specifically K^+^, Na^+^, and Ca^2+^. Organic urinary constituents, such as creatinine, urea, and uric acid, are treated as molecular targets rather than ions and are widely reported to be detected using enzymatic or non-enzymatic electrochemical sensors based on specific redox or catalytic mechanisms, rather than ion-selective membrane systems [37]. The current sensor functions as a chemical ISE governed by ion-ionophore interactions; direct interference from these organic species is expected to be minimal. The $K_{ij}$ value measurements indicated that a $K_{ij}$ < 1 signifies that the Mg^2+^ ion sensor’s response is preferential toward Mg^2+^ relative to the other similar ions within the investigated concentration range. The order of $K_{ij}$ values determined in this study is Ca^2+^ > K^+^ > Na^+^ (see Table S1).

### 3.4. ISE’s Studies on Stability

#### 3.4.1. Evaluation of Response Time

**Figure 5:**
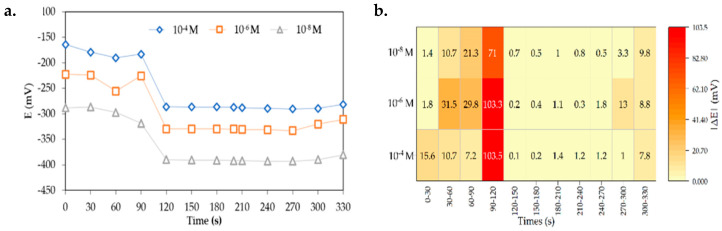
Potential response of the Mg^2+^|M8I2+T ISE. Response time (**a**); Potential differential (∆E) stability (**b**).

The initial stability of the Mg^2+^|M8I2+T ISE was evaluated by measuring the response time through potential recordings taken every 30 s for all tested Mg^2+^ ion concentrations (Figure 5a). A potential differential (∆E) stability may be achieved within the range of 0.1–1.8 mV between 120 and 270 s (Figure 5b). The findings are consistent with the International Union of Pure and Applied Chemistry (IUPAC) standard for potential stability limits (±1 mV) [19,38] and validate analytical assessments conducted after 120 s [39].

Potential ISE data ranging from 120 to 270 s indicate a delay in achieving membrane interface equilibrium, essential for preventing simultaneous activities that could cause drift, a common problem in solid-contact (SC) ISE [40]. The drift is mostly caused by water penetration or deficiencies in the contact between the electron-conducting membrane and the interface. The behaviors are the same for all concentrations of Mg^2+^ ions: 10^−4^ M, 10^−6^ M, and 10^−8^ M.

The pattern proves the efficiency of the ion transport system properties, even at concentrations close to the LoD. XRD results reveal that the morphological design of membrane materials creates free volume for ion diffusion by promoting molecular transport across the interstitial gaps among polymer chains [41]. The uniform distribution of D2EHPA in this domain facilitates the attainment of potential equilibrium. The membrane’s thick and smooth surface characteristics, as evidenced by SEM data, enhance the stability of the membrane–solution interface, hence expediting the stabilization of potential responses and promoting the stability of future reactions.

#### 3.4.2. Reproducibility and Repeatability

**Figure 6:**
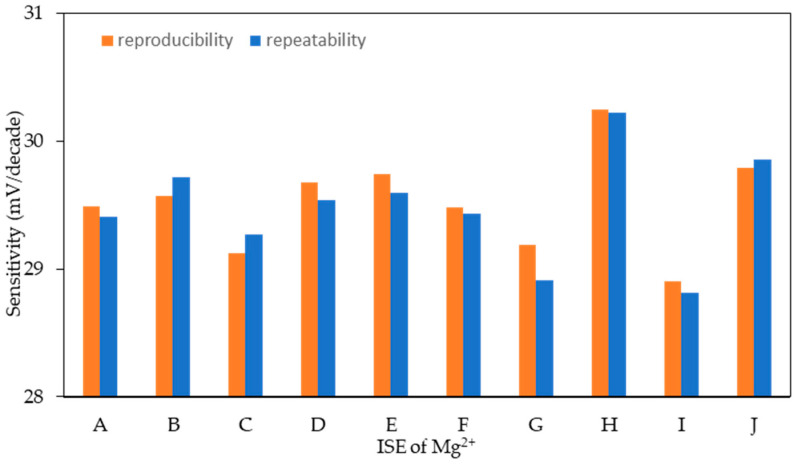
The reproducibility and repeatability profile of the Mg^2+^|M8I2+T ISE.

The reproducibility and repeatability of ten batches of Mg^2+^|M8I2+T ISE (A–J) were evaluated based on Nernstian values (Figure 6), all exhibiting sensitivity within the appropriate range for divalent ions of 28.5–30.5 mV/decade, as presented in SI; see Table S2. The average sensitivity value was 29.52 mV/decade with %RSD of 1.31%, indicating excellent repeatability. Simultaneously, the repeatability yielded an average sensitivity of 29.48 mV/decade, with an average SD of 0.15 mV/decade and a %RSD of 0.50. Both outcomes indicate the electrode’s advantageous short-term stability.

Sensitivities of the ISE’s D and E are close to theoretical Nernstian values (29.58 mV/decade), which indicates an exceptional performance. Conversely, the ISE of G and I demonstrated increased variability that is perhaps attributable to a less homogeneous distribution of membrane components. Repeatability refers to the stability of response under a single, constant condition, whereas reproducibility denotes the uniformity of response across several electrode utilization cycles, as defined by IUPAC [38].

The hydrophobic modified polyurethane membrane is engineered for use in a liquid-contact internal solution system. This design ensures membrane dimensional stability and promotes a homogeneous distribution of D2EHPA throughout the membrane matrix. D2EHPA functions as the selective ionophore for Mg^2+^ ions [20], whereas κ-carrageenan serves not merely as a structural modifier but as a sulfated polysaccharide that introduces sulfate-rich domains, enhancing the membrane’s negative charge density and promoting electrostatic attraction of divalent cations [25]. The corresponding PU membrane modifiers were listed accordingly (see Table 4). As highlighted by the dotted-line boxes, κ-carrageenan is covalently integrated into the polyurethane matrix through urethane bond formation between hydroxyl groups of κ-carrageenan and isocyanate groups of TDI. This process involves partial disruption and reorganization of intermolecular hydrogen bonds, leading to a stable cross-linked network. Importantly, the sulfate (SO_4_^2−^) groups of κ-carrageenan remain ionically active, providing electrostatic stabilization and charge balance within the membrane phase. In parallel, D2EHPA interacts with Mg^2+^ ions via dimer formation, where hydrogen-bonded D2EHPA dimers are replaced by metal–oxygen coordination involving phosphoryl oxygen atoms. This coordination-driven interaction governs selective Mg^2+^ ion recognition and facilitated ion transport, while the hydrophobic alkyl chains ensure compatibility with the PU matrix and suppress ionophore leaching.

In this approach, the hydrophobic characteristic is not intended to obstruct the water layer as observed in SC electrodes but instead to stabilize the membrane environment and diminish the release of ionophores [34]. These results align with Cheong et al. [40], highlighting the role of controlled charge distribution in improving ISE reproducibility.

#### 3.4.3. Functional Lifetime

**Figure 7:**
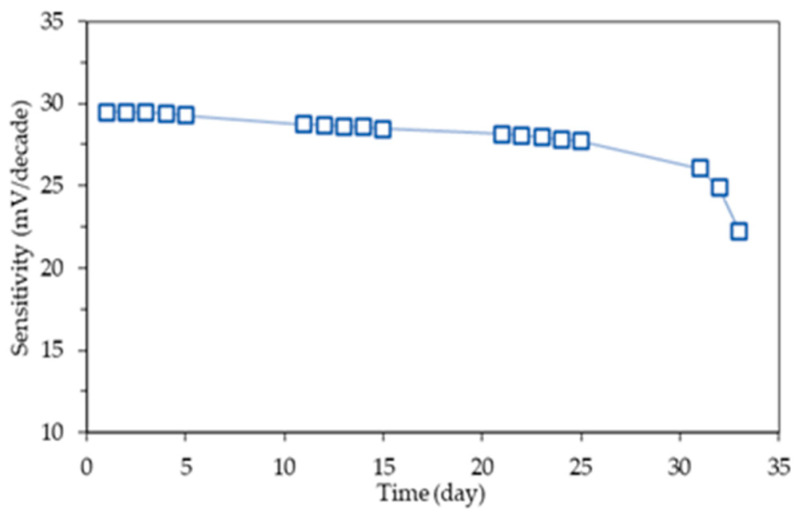
Mg^2+^|M8I2+T ISE functional lifetime profile.

The electrodes’ stability over an extended duration of 33 days was monitored (Figure 7). The curve’s slope remained stable around 28–30 mV/decade until day 28, after which it decreased to 26.07 mV/decade on day 31. The electrode’s functional lifetime constraints become apparent on the 32nd day, as it falls below the theoretical minimum threshold of 24.58 mV/decade [38].

The membrane material’s design profoundly affects its performance. The amorphous structure provides flexibility that improves initial stability; nevertheless, extended exposure may result in polymer relaxation and alterations in molecular locations, potentially affecting conductivity and ionophore–ion interactions of the target. The hydrophobic properties of the PU membrane enhance phase stability and prevent degradation processes linked to swelling or leaching. SEM imaging illustrating solid shape without open porosity can enhance ionophore retention and maintain structural integrity.

Research demonstrates that the integration of solid morphology, uniform amorphous structure, and uniformly distributed active components results in membrane resistance lasting up to 31 days of operation. This endurance exceeds PU membranes reported in previous studies [18] and matches the performance of SC-ISE, although with greater fabrication complexity [40,42]. Furthermore, the applied storage procedure is critical for preserving the extended functional lifetime. Unused ISE was kept in dried condition to avoid ionophore leaching during idle periods.

### 3.5. Recovery Study for Magnesium Ion Sensing

The recovery performance of the Mg^2+^ ISE was evaluated using standard solutions at concentrations of 10^−4^, 10^−6^, and 10^−7^ M. The results are presented in SI; see Figure S2. The recovery percentages range from 98.65% to 104.35%—comparable to those reported in previous studies on solid-state ISEs [32]. According to the U.S. FDA guidelines [43], acceptable recovery values for bioanalytical methods fall within the range of 85% to 115%. Based on this standard, the Mg^2+^ ISE is accurate and suitable for the quantitative determination of magnesium ions.

Table 5 presents a comparative summary of the analytical performance of the developed Mg^2+^ ISE and previously reported sensors. The new proposed ISE exhibits a near-Nernstian slope of 29.49 ± 0.01 mV·dec^−1^, a wide linear range of 10^−9^–10^−4^ M, and a lower LoD, surpassing most earlier Mg^2+^ ISEs. The enhanced performance is attributed to the synergistic interaction in the modified PU membrane, which improves ion exchange and membrane stability. Overall, the ISE demonstrates better selectivity, reproducibility, and operational stability, confirming its suitability for precise Mg^2+^ ion sensing.

## 4. Conclusions

A novel potentiometric ISE based on a castor oil-derived polyurethane (PU) membrane modified with κ-carrageenan and D2EHPA was successfully developed to selectively and sensitively detect magnesium ions. The optimized membrane composition enabled a Nernstian response of 29.49 ± 0.01 mV/decade over a wide linear range, demonstrating excellent correlation. The ISE had a relatively low LoD, a response time of 200 s, and good operational stability across the pH range of 6–8, maintaining stability for up to a month. The characterization results confirmed the successful integration of modifiers into the PU matrix, giving it an amorphous structure and a compact morphology. The ISE showed a high selectivity against common interfering ions. The recovery range confirmed its analytical reliability. Overall, the modified PU membrane-based ISE is a promising and effective platform for the quantitative determination of magnesium ions in aqueous samples.

## Figures and Tables

**Table 1:** Comparison of the modified membrane composition variations on the performance of the Mg^2+^ ISEs.

No.	Membrane Composition (%*w*/*w*)					Sensitivity (mV/Decade)	Linear Range (M)	$R^{2}$
	Matrix				Solvent			
	Castor Oil	TDI	ĸ- Carrageenan	D2EHPA	Acetone			
1	42.4242	21.2121	0	0	36.3636	14.47 ± 0.65	10^−3^–10^−1^	0.9771
2	42.4240	21.2120	0	0.0006	36.3634	11.50 ± 0.09	10^−6^–10^−1^	0.8476
3	42.4237	21.2119	0	0.0012	36.3632	9.71 ± 0.35	10^−4^–10^−1^	0.9450
4	42.4237	21.2119	0.0012	0	36.3632	5.80 ± 0.05	10^−10^–10^−8^	0.9372
5	42.4217	21.2108	0.0061	0	36.3614	5.10 ± 0.25	10^−4^–10^−1^	0.7807
6	42.4235	21.2117	0.0012	0.0006	36.3630	23.51 ± 0.14	10^−6^–10^−3^	0.9918
7	42.4232	21.2116	0.0012	0.0012	36.3628	21.49 ± 0.04	10^−6^–10^−3^	0.9648
8	42.4214	21.2107	0.0061	0.0006	36.3612	^a^ 29.49 ± 0.01	^b^ 10^−9^–10^−4^	^c^ 0.9919
9	42.4212	21.2106	0.0061	0.0012	36.3610	28.15 ± 0.05	10^−7^–10^−3^	0.9997
10	42.4204	21.2102	0.0085	0.0006	36.3603	26.33 ± 0.03	10^−6^–10^−2^	0.9886
11	42.4201	21.2101	0.0085	0.0012	36.3601	22.25 ± 0.08	10^−5^–10^−2^	0.9992
12	42.4188	21.2094	0.0121	0.0006	36.3590	18.65 ± 0.06	10^−4^–10^−1^	0.9363
13	42.4186	21.2093	0.0121	0.0012	36.3588	15.27 ± 0.1	10^−5^–10^−2^	0.9770

a, b, and c represent the performance of the optimized membrane.

**Table 2:** Internal solution concentration effect on the response of the Mg^2+^ ISE.

No.	Internal Solution Composition (M)		Sensitivity (mV/Decade)	Linear Range (M)	$R^{2}$
	Mg(NO_3_)_2_	KCl			
1	0	**0.1**	23.41 ± 0.05	10^−9^–10^−6^	0.9845
**2**	**0.1**		**29.49 ± 0.01**	**10^−9^**–**10^−4^**	**0.9919**
3	0.3		31.17 ± 0.03	10^−9^–10^−5^	0.9018
4	0.5		27.58 ± 0.02	10^−9^–10^−5^	0.9841
5	0.7		30.28 ± 0.05	10^−9^–10^−5^	0.9682

**Table 3:** TISAB effect on the response of the Mg^2+^ ISE.

No.	TISAB Variation	Sensitivity (mV/Decade)	Linear Range (M)	$R^{2}$
**1**	**+TISAB**	**29.49 ± 0.01**	**10^−9^**–**10^−4^**	**0.9919**
2	−TISAB	28.32 ± 0.02	10^−8^–10^−4^	0.9994

**Table 4:** PU membrane modifiers and their optimized molecular structures within the Mg^2+^ ISE system.

Membrane Modifier and Interaction	Proposed Optimized Molecular Structure (Created in Chemcraft 1.8 (Build 748b))
κ-carrageenan–TDI cross-linking network via urethane bond formation [24,25,26]	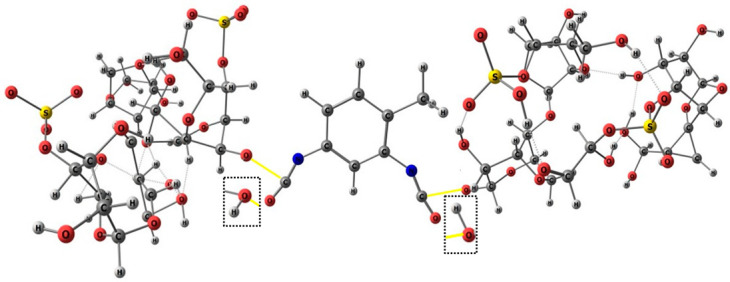
D2EHPA–Mg^2+^ ion coordination complex via dimer formation [28]	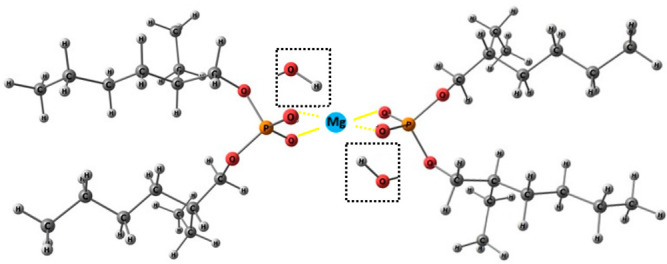

**Table 5:** Analytical performance comparison of Mg^2+^ ISEs.

Material and Active Compound	Sensitivity (mV/Decade)	Linear Range (M)	LOD (M)	Reference
Esomeprazole magnesium trihydrate–carbon–NPOE	29.93 ± 0.1	1.41 × 10^−5^–1 × 10^−2^	4.13 × 10^−6^	[10]
Poly(Tetrahydrofurfuryl Acrylate) (pTHFA) thin-film photopolymer	26.9	0.1–1 × 10^−6^	7.6 × 10^−7^	[11]
Benzo-15-crown-5 (PVC membrane)	31–39	1 × 10^−5^–1 × 10^−1^	—	[44]
ETHT 5504 (neutral carrier) in PVC membrane microelectrode	28.6–30.2	~1 × 10^−7^–1 × 10^−3^	~8 × 10^−6^	[45]
Quetiapine-based ionophore	30.2	1 × 10^−7^–1 × 10^−2^	8 × 10^−8^	[46]
Polypyrrole/Titan Yellow (PGE/PPy/TY)	28.27 ± 0.40	1 × 10^−5^–5 × 10^−2^	6.28 × 10^−6^	[47]
Magnesium salicylate (PVC membrane, heterogeneous precipitate)	23–28	1 × 10^−5^–1 × 10^−1^	—	[48]
Methyl phenyl semicarbazone (PVC membrane)	28.4	1 × 10^−6^–1 × 10^−1^	6.3 × 10^−7^	[49]
4,5-Bis(benzoylthio)-1,3-dithiole-2-thione (Bz_2_dmit, PVC membrane)	29.2	1 × 10^−5^–1 × 10^−1^	1 × 10^−5^	[50]
ETH 1117/ETH 5504/ETH 5506 (Mg^2+^-selective microelectrode series)	—	Micromolar range	~1 × 10^−6^	[51]
PU/κ-carrageenan/D_2_EHPA	29.49 ± 0.01	1 × 10^−9^–1 × 10^−4^	1.25 × 10^−10^	The Present Work

## Data Availability

The data supporting the findings of this study are available from the corresponding author upon reasonable request.

## Supplementary Materials

The following are available online at https://www.mdpi.com/article/10.3390/bios16010055/s1, Table S1: Selectivity coefficient of Mg^2+^|M8I2+T ISE using the separate solution method. Table S2: Repeatability and reproducibility test of Mg^2+^ ISE. Figure S1: Potentiometric response of Mg^2+^|M8I2+T ISE in five repetitions for LoD and LoQ analysis. Figure S2: Recovery performance of the Mg^2+^ ISE.

## References

[1] Jomova K., Makova M., Alomar S.Y., Alwasel S.H., Nepovimova E., Kuca K., Rhodes C.J., Valko M. (2022). Essential Metals in Health and Disease. Chem. Biol. Interact..

[2] Escobedo-Monge M.F., Barrado E., Parodi-Román J., Escobedo-Monge M.A., Marcos-Temprano M., Marugán-Miguelsanz J.M. (2022). Magnesium Status and Calcium/Magnesium Ratios in a Series of Cystic Fibrosis Patients. Nutrients.

[3] Maier J.A.M., Locatelli L., Fedele G., Cazzaniga A., Mazur A. (2022). Magnesium and the Brain: A Focus on Neuroinflammation and Neurodegeneration. Int. J. Mol. Sci..

[4] Escobedo-Monge M.F., Barrado E., Parodi-Román J., Escobedo-Monge M.A., Torres-Hinojal M.C., Marugán-Miguelsanz J.M. (2022). Magnesium Status and Ca/Mg Ratios in a Series of Children and Adolescents with Chronic Diseases. Nutrients.

[5] Guerrero-Romero F., Mercado M., Rodríguez-Morán M., Ramírez-Renteria C., Martínez-Aguilar G., Marrero-Rodríguez D., Ferreira-Hermosillo A., Simental-Mendía L.E., Remba-Shapiro I., Gamboa-Gómez C.I. (2022). Magnesium-to-Calcium Ratio and Mortality from COVID-19. Nutrients.

[6] Costello R.B., Elin R.J., Rosanoff A., Wallace T.C., Guerrero-Romero F., Hruby A., Lutsey P.L., Nielsen F.H., Rodriguez-Moran M., Song Y. (2016). Perspective: The Case for an Evidence-Based Reference Interval for Serum Magnesium: The Time Has Come. Adv. Nutr..

[7] Lvova L., Gonçalves C.G., Di Natale C., Legin A., Kirsanov D., Paolesse R. (2018). Recent Advances in Magnesium Assessment: From Single Selective Sensors to Multisensory Approach. Talanta.

[8] Fiorentini D., Cappadone C., Farruggia G., Prata C. (2021). Magnesium: Biochemistry, Nutrition, Detection, and Social Impact of Diseases Linked to Its Deficiency. Nutrients.

[9] Dent A., Selvaratnam R. (2022). Measuring Magnesium—Physiological, Clinical and Analytical Perspectives. Clin. Biochem..

[10] Alva S., Oktavianus Y., Sundari R., Aziz A.S.A., Hasbullah S.A. (2024). Fabrication of a New Magnesium-Ion Selective Electrode (Mg-ISE) Based on Photopolymer of Poly(Tetrahydrofurfuryl Acrylate) (pTHFA) Thin-Films. Mater. Today Proc..

[11] Saad A.S., Ismail N.S., Gaber N.S., Elzanfaly E.S. (2023). A Chemically Modified Solid-State Sensor for Magnesium(II) Ions and Esomeprazole Magnesium Potentiometric Assay. RSC Adv..

[12] Kabaa E.A., Abdulateef S.A., Ahmed N.M., Hassan Z., Sabah F.A. (2019). A Novel Porous Silicon Multi-Ions Selective Electrode Based Extended Gate Field Effect Transistor for Sodium, Potassium, Calcium, and Magnesium Sensor. Appl. Phys. A Mater. Sci. Process..

[13] Mele L.J., Palestri P., Alam M.A., Selmi L. (2022). Selectivity, Sensitivity and Detection Range in Ion-Selective Membrane-Based Electrochemical Potentiometric Sensors Analyzed With Poisson-Boltzmann Equilibrium Model. IEEE Sens. J..

[14] Nygaard T.P., Schneider P., Neilson P.J., Hansen T.S., Laursen T. (2021). Magnesium Ion Selective Membranes. CN Patent.

[15] Maksymiuk K., Stelmach E., Michalska A. (2020). Unintended Changes of Ion-Selective Membranes Composition—Origin and Effect on Analytical Performance. Membranes.

[16] Kazemi P., Peydayesh M., Bandegi A., Mohammadi T., Bakhtiari O. (2013). Pertraction of Methylene Blue Using a Mixture of D2EHPA/M2EHPA and Sesame Oil as a Liquid Membrane. Chem. Pap..

[17] Baig U., Khan A.A. (2015). Polyurethane-Based Cation Exchange Composite Membranes: Preparation, Characterization and Its Application in Development of Ion-Selective Electrode for Detection of Copper(II). J. Ind. Eng. Chem..

[18] Safitri E., Nazaruddin N., Humaira R., Afifi M.R., Saleha S., Sani N.D.M., Idroes R., Ulianas A., Hidayat N., Rumhayati B. (2025). The Construction of Castor Oil (*Ricinus communis* L.) Polyurethane Membrane Embedded 1,10-Phenanthroline Graphene as a Cr 3+ Ion Selective Membrane. J. Electrochem. Soc..

[19] Nisah K., Rahmi R., Ramli M., Idroes R., Alva S., Iqhrammullah M., Safitri E. (2022). Optimization of Castor Oil-Based Ion Selective Electrode (ISE) with Active Agent 1,10-Phenanthroline for Aqueous Pb^2+^ Analysis. Membranes.

[20] Azarang A., Rahbar-Kelishami A., Norouzbeigi R., Shayesteh H. (2019). Modeling and Optimization of Pertraction Performance of Heavy Metal Ion from Aqueous Solutions Using M2EHPA/D2EHPA: Application of Response Surface Methodology. Environ. Technol. Innov..

[21] Hanum F., Safitri E., Idroes R., Idris N. (2024). Applying Box-Behnken Experimental Design for Optimization of Polyurethane Membrane Synthesis from Castor Oil (*Ricinus communis* L.) Based on Physical Performance. Mater. Sci. Forum.

[22] Qin W., Liang R., Fu X., Wang Q., Yin T., Song W. (2012). Trace-Level Potentiometric Detection in the Presence of a High Electrolyte Background. Anal. Chem..

[23] Phoonsawat K., Ozer T., Dungchai W., Henry C.S. (2022). Dual-Mode Ion-Selective Electrodes and Distance-Based Microfluidic Device for Detection of Multiple Urinary Electrolytes. Analyst.

[24] Whitehouse R.S. (2014). Polyurethanes Obtained from Hydroxyalkanoate Crosslinking Agents.

[25] Okolišan D., Vlase G., Vlase T., Avram C. (2022). Preliminary Study of κ-Carrageenan Based Membranes for Anti-Inflammatory Drug Delivery. Polymers.

[26] Papageorgiou M., Nanaki S.G., Kyzas G.Z., Koulouktsi C., Bikiaris D.N., Lambropoulou D.A. (2017). Novel Isocyanate-Modified Carrageenan Polymer Materials: Preparation, Characterization and Application Adsorbent Materials of Pharmaceuticals. Polymers.

[27] Nurman S., Saiful S., Ginting B., Rahmi R., Marlina M., Wibisono Y. (2021). Synthesis of Polyurethane Membranes Derived from Red Seaweed Biomass for Ammonia Filtration. Membranes.

[28] Biswas R.K., Habib M.A., Islam M.N. (2000). Some Physicochemical Properties of (D2EHPA). 1. Distribution, Dimerization, and Acid Dissociation Constants of D2EHPA in a Kerosene/0.10 Kmol m^−3^ (Na^+^,H^+^)Cl^−^ System and the Extraction of Mn(II). Ind. Eng. Chem. Res..

[29] Zahid M., Saeeda M., Nadeem N., Shakir H.M.F., El-Saoud W.A., Attala O.A., Attia K.A., Rehan Z.A. (2023). Carboxylated Graphene Oxide (c-GO) Embedded ThermoPlastic Polyurethane (TPU) Mixed Matrix Membrane with Improved Physicochemical Characteristics. Membranes.

[30] Caddeo C., Marongiu D., Meloni S., Filippetti A., Quochi F., Saba M., Mattoni A. (2019). Hydrophilicity and Water Contact Angle on Methylammonium Lead Iodide. Adv. Mater. Interfaces.

[31] Mani M.P., Mohanadas H.P., Faudzi A.A.M., Ismail A.F., Tucker N., Mohamaddan S., Ayyar M., Palanisamy T., Rathanasamy R., Jaganathan S.K. (2024). Characterization and Performance Evaluation of Magnesium Chloride-Enriched Polyurethane Nanofiber Patches for Wound Dressings. Int. J. Nanomed..

[32] Chen L.-D.D., Wang W.-J.J., Wang G.-J.J. (2021). Electrochemical Detection of Electrolytes Using a Solid-State Ion-Selective Electrode of Single-Piece Type Membrane. Biosensors.

[33] Ito T., Yamazaki M. (2006). The “Le Chatelier’s Principle”-Governed Response of Actin Filaments to Osmotic Stress. J. Phys. Chem. B.

[34] Nisah K., Safitri E., Rahmi R., Ramli M., Nasution R.S., Iqhrammullah M. (2024). Ion-Selective Electrode Based on Polyurethane-Immobilized Di-(2-Ethyl Hexyl) Phosphoric Acid for Low-Concentration Aqueous Pb2+ Detection and Quantification. Biosens. Bioelectron. X.

[35] Shi D., Cui B., Li L., Xu M., Zhang Y., Peng X., Zhang L., Song F., Ji L. (2020). Removal of Calcium and Magnesium from Lithium Concentrated Solution by Solvent Extraction Method Using D2EHPA. Desalination.

[36] Abbaspour A., Izadyar A. (2001). Chromium(III) Ion-Selective Electrode Based on 4-Dimethylaminoazobenzene. Talanta.

[37] Gonzalez-Gallardo C., Arjona N., Álvarez-Contreras L., Guerra-Balcázar M. (2022). Electrochemical Creatinine Detection for Advanced Point-of-Care Sensing Devices: A Review. RSC Adv..

[38] Buck R.P., Lindner E. (1994). Recomendations for Nomenclature of Ion-Selective Electrodes (IUPAC Recommendations 1994). Pure Appl. Chem..

[39] Maccà C. (2004). Response Time of Ion-Selective Electrodes: Current Usage versus IUPAC Recommendations. Anal. Chim. Acta.

[40] Cheong Y.H., Ge L., Lisak G. (2021). Highly Reproducible Solid Contact Ion Selective Electrodes: Emerging Opportunities for Potentiometry—A Review. Anal. Chim. Acta.

[41] Çelik G., Barsbay M., Güven O. (2016). Towards New Proton Exchange Membrane Materials with Enhanced Performance via RAFT Polymerization. Polym. Chem..

[42] Faridbod F., Ganjali M.R., Dinarvand R., Norouzi P. (2008). Developments in the Field of Conducting and Non-Conducting Polymer Based Potentiometric Membrane Sensors for Ions over the Past Decade. Sensors.

[43] U.S. Department of Health and Human Services, Food and Drug Administration, Center for Drug Evaluation and Research (CDER), Center for Veterinary Medicine (CVM) (2018). Bioanalytical Method Validation Guidance for Industry. https://www.fda.gov/files/drugs/published/Bioanalytical-Method-Validation-Guidance-for-Industry.pdf.

[44] Gupta V.K., Chandra S., Mangla R. (2002). Magnesium-Selective Electrodes. Sens. Actuators B Chem..

[45] Zhang X., Fakler A., Spichiger U.E. (1998). Development of Magnesium-Ion-Selective Microelectrodes Based on a New Neutral Carrier ETHT 5504. Electroanal. Int. J. Devoted Fundam. Pract. Asp. Electroanal..

[46] Sarvestani M.R.J., Doroudi Z. (2021). Determination Trace Amounts of Magnesium (II) by a Potentiometric Sensor Based on Quetiapine as an Ionophore. Chem. Asian J..

[47] Mosayebzadeh Z., Ansari R., Arvand M. (2014). Preparation of a Solid-State Ion-Selective Electrode Based on Polypyrrole Conducting Polymer for Magnesium Ion. J. Iran. Chem. Soc..

[48] Sudha M., Selvi J. (2014). A Heterogeneous Precipitate Based Magnesium Ion Selective Electrode-Its Preparation and Analytical Application. Orient. J. Chem..

[49] Chandra S., Sharma K., Kumar A. (2013). Mg(II) Selective PVC Membrane Electrode Based on Methyl Phenyl Semicarbazone as an Ionophore. J. Chem..

[50] Zamani H.A., Nezhadali A., Saghravanian M. (2008). Magnesium-PVC Membrane Sensor Based on 4,5-Bis(Benzoylthio)-1,3-Dithiole-2-Thione. Anal. Lett..

[51] Günzel D., Schlue W.-R. (2002). Determination of [Mg^2+^]i—An Update on the Use of Mg^2+^-Selective Electrodes. Biometals.

