# Supplementary material for: Enhanced Magnesium Ion Sensing Using Polyurethane Membranes Modified with ĸ-Carrageenan and D2EHPA: A Potentiometric Approach
Source: Biosensors (Basel). 2026 Jan 12;16(1):55. doi: 10.3390/bios16010055 (PMC12839118; doi:10.3390/bios16010055)

Supporting Information

Enhanced Magnesium Ion Sensing Using Polyurethane Membranes Modified with κ-Carrageenan and D2EHPA: A Potentiometric Approach

Faridah Hanum [1,2], Salfauqi Nurman [3], Nurhayati [4], Nasrullah Idris [5] Rinaldi Idroes [6,7], and Eka Safitri [7,*]

[1] Graduate School of Mathematics and Applied Sciences, Universitas Syiah Kuala, Banda Aceh 23111, Indonesia; faridah63@mhs.usk.ac.id

[2] Department of Pharmacy, Poltekkes Kemenkes Aceh, Aceh Besar, 23231, Indonesia; hanum_jamu@poltekkesaceh.ac.id

[3,4] Department of Ship Engineering, Politeknik Pelayaran Malahayati, Aceh Besar 23381, Indonesia

[5] Department of Physics, Faculty of Mathematics and Natural Sciences, Universitas Syiah Kuala, Banda Aceh 23111, Indonesia; nasrullah.idris@usk.ac.id

[6] Department of Pharmacy, Faculty of Mathematics and Natural Sciences, Universitas Syiah Kuala, Banda Aceh 23111, Indonesia; rinaldi.idroes@usk.ac.id

[7] Department of Chemistry, Faculty of Mathematics and Natural Sciences, Universitas Syiah Kuala, Banda Aceh 23111, Indonesia; e.safitri@usk.ac.id

* Correspondence: e.safitri@usk.ac.id

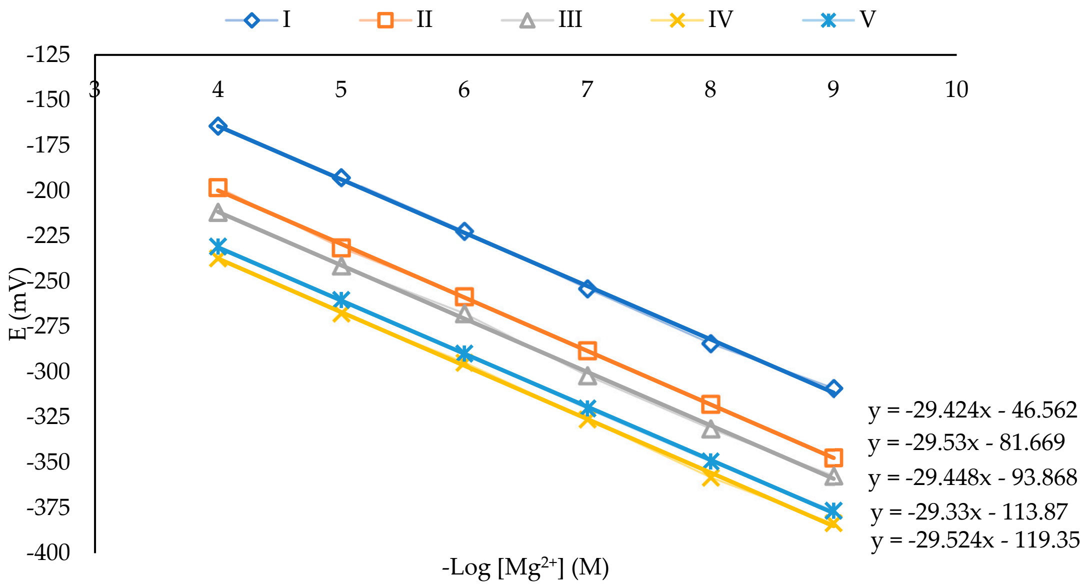

Figure S1. Potentiometric response of Mg^{2+}|M8I2+T ISE in five repetitions for LoD and LoQ analysis.

Table S1. Selectivity coefficient of Mg^{2+}|M8I2+T ISE using the separate solution method.

i [Mg^{2+}]	j [K^+]	[i]	[j]	E_i (mv)			E_i	E_j	ΔE (E_j-E_i)	S	γ_i	γ_j	α_i	α_j	ΔE/S(j-i)	K_{ij} (j-i)	-Log K_{ij}
**.10^{-4}**	.10^{-4}	0.0001	0.0001512	-163.8	-165.3	-163.7	-164.3	-103.5	60.8	29.42	0.022235	0.006219	2.224E-06	6E-07	2.06549	3.28E-01	**0.48**
**.10^{-4}**	.10^{-5}	0.0001	0.00001512	-192.5	-194.4	-191.6	-164.3	-105.7	58.6	29.42	0.022235	0.001983	2.224E-06	2E-08	1.99071	1.55E+00	-0.19
**.10^{-4}**	.10^{-6}	0.0001	0.000001512	-222.3	-224.1	-220.8	-164.3	-108.3	56.0	29.42	0.022235	0.000629	2.224E-06	6E-10	1.90233	7.08E+00	-0.85
**.10^{-4}**	.10^{-7}	0.0001	1.512E-07	-259.8	-252.6	-249.8	-164.3	-111.6	52.7	29.42	0.022235	0.000199	2.224E-06	2E-11	1.79017	3.07E+01	-1.49
**.10^{-4}**	.10^{-8}	0.0001	1.512E-08	-288.1	-284.8	-280.1	-164.3	-115.2	49.1	29.42	0.022235	6.29E-05	2.224E-06	6E-13	1.6678	1.30E+02	-2.12
**.10^{-4}**	.10^{-9}	0.0001	1.512E-09	-305.4	-311.5	-310.1	-164.3	-119.4	44.9	29.42	0.022235	1.99E-05	2.224E-06	2E-14	1.52504	5.28E+02	-2.72
**i [Mg^{2+}]**	**j [K^+]**	**[i]**	**[j]**	**E_i (mv)**			**E_i**	**E_j**	**ΔE (E_j-E_i)**	**S**	**γ_i**	**γ_j**	**α_i**	**α_j**	**ΔE/S(j-i)**	**K_{ij} (j-i)**	**-Log K_{ij}**
**.10^{-4}**	.10^{-4}	0.0001	0.0001512	-163.8	-165.3	-163.7	-164.3	-104.1	60.2	29.42	0.022235	0.006219	2.224E-06	6E-07	2.04509	3.13E-01	**0.50**
**.10^{-4}**	.10^{-5}	0.0001	0.00001512	-192.5	-194.4	-191.6	-164.3	-108.6	55.7	29.42	0.022235	0.001983	2.224E-06	2E-08	1.89214	1.23E+00	-0.09
**.10^{-4}**	.10^{-6}	0.0001	0.000001512	-222.3	-224.1	-220.8	-164.3	-113.6	50.7	29.42	0.022235	0.000629	2.224E-06	6E-10	1.72218	4.68E+00	-0.67
**.10^{-4}**	.10^{-7}	0.0001	1.512E-07	-259.8	-252.6	-249.8	-164.3	-119.3	45.0	29.42	0.022235	0.000199	2.224E-06	2E-11	1.52844	1.68E+01	-1.23
**.10^{-4}**	.10^{-8}	0.0001	1.512E-08	-288.1	-284.8	-280.1	-164.3	-122.1	42.2	29.42	0.022235	6.29E-05	2.224E-06	6E-13	1.43327	7.60E+01	-1.88
**.10^{-4}**	.10^{-9}	0.0001	1.512E-09	-305.4	-311.5	-310.1	-164.3	-126.3	38.0	29.42	0.022235	1.99E-05	2.224E-06	2E-14	1.29051	3.08E+02	-2.49
**i [Mg^{2+}]**	**j [K^+]**	**[i]**	**[j]**	**E_i (mv)**			**E_i**	**E_j**	**ΔE (E_j-E_i)**	**S**	**γ_i**	**γ_j**	**α_i**	**α_j**	**ΔE/S(j-i)**	**K_{ij} (j-i)**	**-Log K_{ij}**
**.10^{-4}**	.10^{-4}	0.0001	0.0003048	-163.8	-165.3	-163.7	-164.3	-170.7	-6.4	29.42	0.022235	0.035142	2.224E-06	4E-06	-0.2187	3.82E-01	**0.42**
**.10^{-4}**	.10^{-5}	0.0001	0.00003048	-192.5	-194.4	-191.6	-164.3	-175.6	-11.3	29.42	0.022235	0.011245	2.224E-06	1E-07	-0.3852	8.14E+00	-0.91
**.10^{-4}**	.10^{-6}	0.0001	0.000003048	-222.3	-224.1	-220.8	-164.3	-186.5	-22.2	29.42	0.022235	0.003569	2.224E-06	4E-09	-0.7557	1.09E+02	-2.04
**.10^{-4}**	.10^{-7}	0.0001	3.048E-07	-259.8	-252.6	-249.8	-164.3	-207.4	-43.1	29.42	0.022235	0.00113	2.224E-06	1E-10	-1.4661	6.73E+02	-2.83
**.10^{-4}**	.10^{-8}	0.0001	3.048E-08	-288.1	-284.8	-280.1	-164.3	-213.5	-49.2	29.42	0.022235	0.000357	2.224E-06	4E-12	-1.6735	1.32E+04	-4.12
**.10^{-4}**	.10^{-9}	0.0001	3.048E-09	-305.4	-311.5	-310.1	-164.3	-218.9	-54.6	29.42	0.022235	0.000113	2.224E-06	1E-13	-1.857	2.73E+05	-5.44

Table S2. Repeatability and reproducibility test of Mg^{2+} ISE.

**[Mg^{2+}] (M)**	Potential average (mV) ± SD (6 times) **Repeatability test**										SD between 10 ISE **Reproducibility test**
	ISE A	ISE B	ISE C	ISE D	ISE E	ISE F	ISE G	ISE H	ISE I	ISE J	
**10^{-4}**	-211.1±0.32	-210.7±0.39	-212.7±0.50	-210.7±0.46	-211.9±0.59	-211±0.41	-210±0.74	-210.3±0.73	-212±1.11	-208.3±0.62	±0.23
**10^{-5}**	-240.5±0.58	-239.6±0.57	-240.5±0.42	-241.1±0.57	-239.6±0.45	-242.7±0.71	-239.2±0.65	-238.2±0.85	-238.3±0.69	-241.7±1.27	±0.24
**10^{-6}**	-274.6±0.37	-272.8±0.63	-273.6±0.59	-272.1±0.43	-273.2±0.47	-274.2±0.38	-272±0.75	-274.2±1.2	-273.4±1.52	-271.7±1.39	±0.44
**10^{-7}**	-301.9±0.89	-300.6±0.48	-299.5±0.66	-300.4±0.50	-299.8±0.37	-301.3±0.31	-297.9±0.91	-302.5±0.76	-300.4±1.43	-299.9±0.34	±0.35
**10^{-8}**	-335.6±0.43	-334.8±0.51	-333.7±0.50	-329.8±0.35	-330.1±0.44	-331.1±0.77	-329.9±0.79	-335±1.1	-327.4±1.37	-333.8±0.65	±0.33
**10^{-9}**	-354.5±0.31	-356±0.69	-356.5±0.28	-358.6±0.27	-359.5±0.48	-358.5±0.56	-352.8±0.97	-358.2±0.91	-354.9±1.55	-356.4±0.68	±0.39
**Sensitivity (mV/dec)**	29.41	29.72	29.27	29.54	29.60	29.43	28.91	30.23	28.82	29.86	Average= 29.48<br>SD= 0.42<br>%RSD=1.43

Figure S2. Recovery performance of the Mg^{2+} ISE.

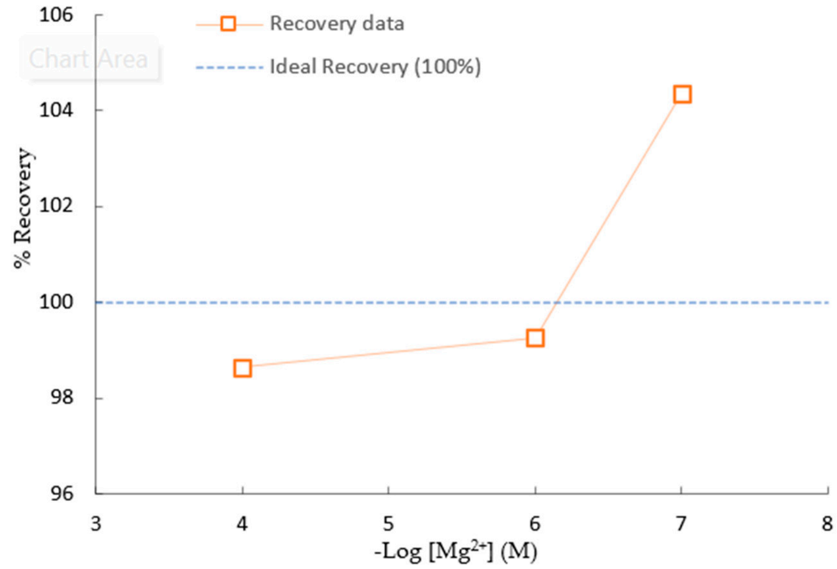

Supplement: Supplementary file 1 [file biosensors-16-00055-s001.zip › biosensors-4028431-supplementary.pdf]
